# Modified tarsorrhaphy versus gold weight implant technique for paralytic lagophthalmos treatment in patients with leprosy: One-year observation of a randomized controlled trial study

**DOI:** 10.3389/fmed.2022.941082

**Published:** 2023-01-04

**Authors:** Yunia Irawati, Michelle Eva Rebeca Natalia, Tjahjono D. Gondhowiardjo, Ishandono Dachlan, Hardyanto Soebono

**Affiliations:** ^1^Division of Plastic Reconstructive Surgery, Department of Ophthalmology, Faculty of Medicine, Dr. Cipto Mangunkusumo National Hospital, University of Indonesia, Jakarta, Indonesia; ^2^JEC Eye Hospitals and Clinics, Jakarta, Indonesia; ^3^Department of Ophthalmology, Faculty of Medicine, University of Indonesia, Jakarta, Indonesia; ^4^Division of Cornea and Refractive Surgery, Department of Ophthalmology, Faculty of Medicine, Dr. Cipto Mangunkusumo National Hospital, University of Indonesia, Jakarta, Indonesia; ^5^Division of Plastic and Reconstructive Surgery, Department of Surgery, Faculty of Medicine, Public Health, and Nursing, Dr. Sardjito Hospital, Gadjah Mada University, Yogyakarta, Indonesia; ^6^Department of Dermatology and Venereology, Faculty of Medicine, Public Health, and Nursing, Dr. Sardjito Hospital, Gadjah Mada University, Yogyakarta, Indonesia

**Keywords:** Hansen’s disease, paralytic lagophthalmos, surgical intervention, epitheliopathy, corneal exposure

## Abstract

**Trial design:**

This study was a multicenter, Prospective Randomized Open-label Blinded-Endpoint (PROBE) clinical trial, parallel-group study conducted in Indonesia (three sites).

**Methods:**

The aim of this study was to compare the effectivity and efficiency of modified tarsorrhaphy (MT) and gold weight implant (GWI) techniques in the surgical treatment of paralytic lagophthalmos in patients with leprosy. The study sample consisted of 23 eyes, with 11 eyes in the MT group and the remaining 12 eyes in the GWI group—the control group.

**Results:**

The central eyelid margin distance (lagophthalmos distance) decreased when gentle pressure was applied in the MT (3.09 mm to 0.43 mm) and GWI groups (3.21 mm to 0.83 mm) at postoperative year 1. The Ocular Surface Disease Index score, the tear break-up time, and the Schirmer test without and with anesthesia in the MT and GWI groups showed a *p*-value of > 0.05. Epitheliopathy improvement occurred in 54.55% of the MT group and 58.33% of the GWI group. Corneal sensitivity change in the inferior quadrant of the MT group (50.00 to 51.30 mm) and in the GWI group (49.61 to 52.93 mm) resulted in a *p* > 0.05. Postoperative complications occurred in 15% of patients in the GWI group. In addition, the surgery duration of both techniques was similar. Furthermore, the surgery cost in the MT and GWI groups yielded a *p* < 0.05.

**Conclusion:**

The MT technique is as effective as the GWI technique but more efficient than the GWI technique as a surgical treatment for paralytic lagophthalmos in patients with leprosy.

**Clinical trial registration:**

[www.ClinicalTrials.gov], identifier [NCT0494 4498].

## Introduction

Lagophthalmos is a condition in which eyelids are incapable of closing completely. This condition can be divided into paralytic and cicatricial based on the etiology. Several factors could induce paralytic lagophthalmos, such as idiopathic nerve paralysis (e.g., Bell’s palsy), infection, trauma, or neoplasm. Paralytic lagophthalmos is one of the most common eye disorders in patients with leprosy caused by *Mycobacterium leprae* invasion on the peripheral endings of the facial cranial nerve, which innervates orbicularis muscles of the eyelids. *Mycobacterium leprae* invasion causes disturbance in axon conduction and nerve demyelination. These disruptions are associated with laminin 2 protein and dystroglycan, which can be found in the peripheral nerve endings of CN VII. When *Mycobacterium leprae* is ligated by neuregulin receptors (ErbB2, ERK 1, and ERK 2), nerve inflammatory responses form. If zygomatic and temporalis branches of CN VII are affected, orbicularis oculi muscles will be paralyzed, resulting in paralytic lagophthalmos.

To date, about 200,000 new leprosy cases are diagnosed worldwide every year. In 2019, the highest number of new leprosy cases is from India (114,451), followed by Brazil (27,863) and Indonesia (17,439). The World Health Organization (WHO) stated that leprosy still required global attention; therefore, the WHO implemented Global Leprosy Strategy 2021-2030 (1). Lagophthalmos might cause corneal opacities, which lead to decreasing visual acuity and blindness (2). The WHO grades visual impairment in leprosy as grade 0, no eye problem due to leprosy; grade 1, eye problems due to leprosy, but vision is not severely affected [visual acuity (VA) 6/60 or better; can count fingers at 6 m]; and grade 2, severe visual impairment (VA worse than 6/60; inability to count fingers at 6 m), including lagophthalmos, corneal anesthesia, and iridocyclitis (3).

The presence of blindness along with another form of disability (extremities, such as hands and feet) will significantly reduce the quality of life of patients with leprosy. In order to prevent such conditions, surgical treatment is considered necessary. In this case, the upper eyelid loading technique is the most commonly used surgical procedure for paralytic lagophthalmos, mainly using the gold weight implant (GWI) technique. Although this technique has a high success rate in the management of paralytic lagophthalmos, the GWI has a fairly high complication rate in patients with leprosy, such as implant extrusion found in six of 12 (50%) patients between 3 and 12 months during the observation period (4). The modified tarsorrhaphy (MT) technique could be considered a treatment in leprosy cases. This technique is more simple to perform and can be carried out even in rural areas with limited availability of oculoplastic surgeons. Furthermore, since patients with leprosy in most cases are from a low socioeconomic class, the cost of surgery needs to be considered. The modified tarsorrhaphy technique might be a more accessible technique than the GWI technique to use for paralytic lagophthalmos in patients with leprosy, but the effectivity and efficiency between the two techniques have not been studied before. This study aims to compare the effectivity and efficiency of the MT and GWI techniques as surgical treatment for paralytic lagophthalmos in patients with leprosy.

## Materials and methods

### Study design and sampling

A multicenter Prospective Randomized Open-label, Blinded-Endpoint (PROBE) clinical trial was conducted in three hospitals in Indonesia. The sample size was determined using a two-tailed hypothesis test, which predicted a minimum number of 12 eyes for each group.


$$n=\frac{S\,d^{2}\,{\left(Z_{1-\alpha /2}+Z_{1-\beta }\right)}^{2}}{{\left(\mu_{0}-\mu_{a}\right)}^{2}}$$

$$n=\frac{1.19^{2}\,{\left(1.96+0.842\right)}^{2}}{{\left(1\right)}^{2}}$$

    n=12

where n = total sample, Sd = standard deviation of mean difference (1.19), Z_1–α /2_ = type 1 error (1.96), Z_1–β_ = type 2 error (0.842), and μ_0_ - μ_a_ = mean significant difference between both groups (1 mm represents significance).

Anticipating a dropout, the researchers added 20% of the total sample. Research team members assigned treatment on all samples *via* randomization with a blocking restriction size of 2. A researcher as the oculoplastic surgeon (YI) acknowledged the treatment assigned to each eye based on randomization. Also, the outcomes were measured by three oculoplastic surgeons with equal clinical experiences (HD, TR, and AP), who had been trained to reduce data bias.

The inclusion criteria were patients with paucibacillary (PB)- or multibacillary (MB)-type leprosy with unilateral/bilateral lagophthalmos, who had not undergone eyelid reconstruction, aged 18 years or older, and who could undergo surgery with local anesthesia. The exclusion criteria were patients with acute leprosy reaction (<6 months) and under steroid medication, and patients with an eyelid laxity > 8 mm. The dropout criteria were patients who had not attended follow-up appointments as determined by researchers and patients who resigned during the study period. Patients who were willing to participate in this study were asked to sign a written consent form. Those in the intervention group received the MT, which was carried out in three steps (Yunia technique):

(1) **Levator recession:** This technique involves (a) an aseptic procedure, eyelid skin crease marking, and local anesthesia injection in the upper eyelid and lateral side; (b) skin crease incision and orbicularis dissection until the tarsal plate, and then conjunctival eversion and ballooning; (c) levator recess; and (d) perform lid crease suture.


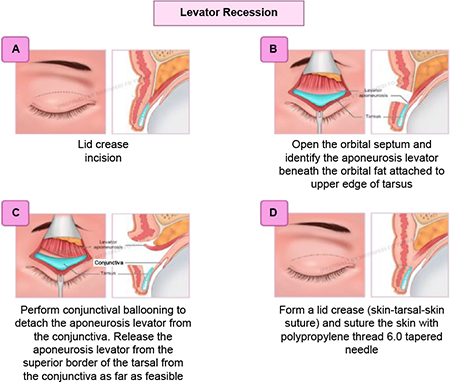


(2) **Lateral tarsorrhaphy:** This technique involves canthotomy, lateral cantholysis, and excision of upper and lower lid margins as long as 10 mm from the lateral canthus to the central area, followed by permanent lateral tarsorrhaphy.


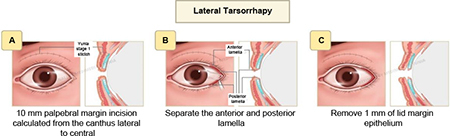


(3) **Canthopexy and canthoplasty or lateral tarsal strip and canthoplasty** were performed according to the horizontal eyelid laxity. The canthopexy/lateral tarsal strip (LTS) used Vicryl 5-0 and canthoplasty with Vicryl 6-0, and (9) skin suture with Prolene 6-0 at skin crease.


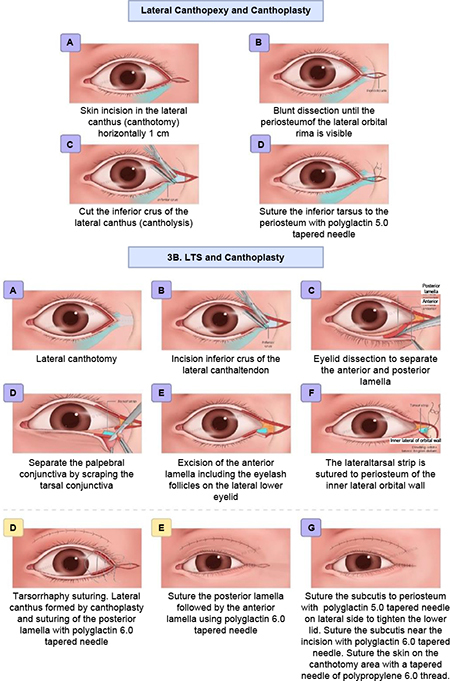


The control group received GWI which was performed with the following steps: (1) aseptic procedure; (2) eyelid skin crease marking; (3) subcutaneous local anesthesia injection in the upper eyelid; (4) skin crease incision and orbicularis dissection; (5) orbital septum opening in the superior tarsal midline; (6) conjunctival eversion and ballooning; (7) placing the gold weight inferior to the levator insertion on the tarsal plate, followed by suturing the implant on the tarsal plate with Prolene 6-0; and (9) suturing pretarsal and preseptal orbicularis muscles with Vicryl 6-0 to cover implant, followed by suturing the skin with Prolene 6-0.

### Data collection

Anticipating a dropout, the researchers added 20% of the total sample. The research team members assigned treatment for all the samples *via* randomization with a blocking restriction size of 2. One of the researchers, an oculoplastic surgeon (YI) acknowledged the treatment assigned to each eye based on randomization. Outcomes were measured by three oculoplastic surgeons with equal clinical experiences (HD, TR, and AP), who had been trained to reduce data bias.

**FIGURE 1:**
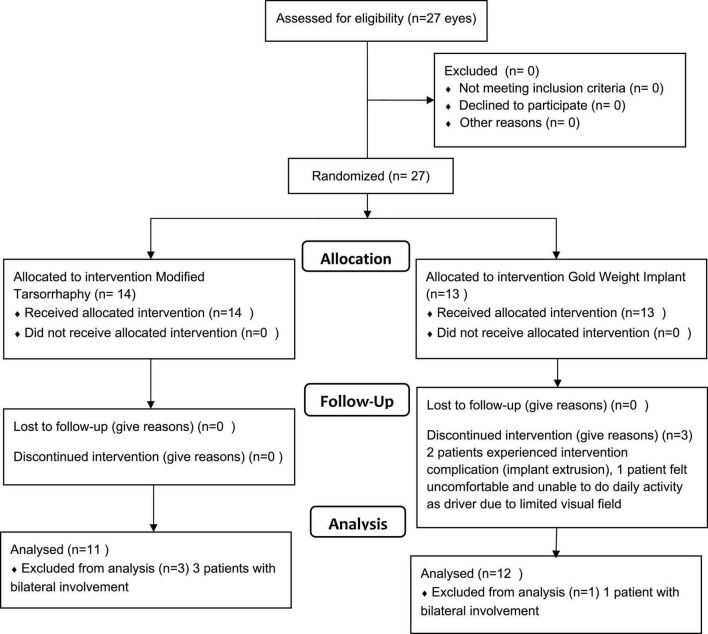
Flow diagram of subject recruitment and group allocation (18–20).

The inclusion criteria were patients with paucibacillary (PB)- or multibacillary (MB)-type leprosy with unilateral/bilateral lagophthalmos who had not undergone eyelid reconstruction, patients aged 18 years or older, and who could undergo surgery with local anesthesia. The exclusion criteria were patients with an acute leprosy reaction (<6 months) and under steroid medication, and patients with an eyelid laxity >8 mm. The dropout criteria were patients who did not attend follow-up appointments as determined by researchers and patients who resigned during the study period. Patients who were willing to participate in this study were asked to sign a written consent form. The total sample was 23 eyes, with 11 eyes in the MT group (intervention group) and 12 eyes in the GWI group (control group) (Figure 1).

### Data analysis

Data were analyzed using the Statistical Package for Social Sciences (SPSS) version 20 developed by International Business Machines (IBM). A univariate analysis was performed to obtain an overview of the frequency distribution. To analyze a normal distribution of data, the Shapiro–Wilk test was used. A bivariate analysis was conducted using an independent *t*-test, the Wilcoxon homogeneity test, and the Mann–Whitney test. In addition, a *p*-value of 0.05 was used as the cutoff point of significance.

### Ethical consideration

For ethical reasons, researchers were blinded to the results. This study was approved by the Medical and Health Research Ethics Committee (MHREC) of the Faculty of Medicine, Public Health and Nursing Universitas Gadjah Mada—Dr. Sardjito General Hospital, Yogyakarta, Indonesia, with the reference number KE/FK/0557/EC/2018 and extended with the reference number KE/FK/0525/EC/2019. This study complies with the 2013 WMA Declaration of Helsinki. Written informed consent was obtained from all the patients after the aim of the study and the nature of their participation were explained to them.

## Results

### Subject and clinical characteristics

A total of 23 eyes from 23 patients were randomly grouped into the MT group (11 eyes) as the intervention group and the GWI group (12 eyes) as the control group. All the participants were observed for 1 year without losses or exclusions after randomization. In this study, 18 patients (78.3%) were male. The patients’ ages ranged from 40 to 77 years (55.45 ± 9.5). Patient characteristics are shown in Table 1.

**TABLE 1:** Patient characteristics.

Patient characteristics	Total patients (*N* = 23)	Percentage (%)
**Age (years)**		
Mean ± SD	(55.45 ± 9.5)	
Range	40–77	
**Sex**		
Male	18	78.3
Female	5	21.7
**Occupation**		
Unemployed	10	43.5
Freelance	13	56.5
**Education level**		
None	9	39.1
Primary School	8	34.8
Junior High School	2	8.7
Senior High School	4	17.4

SD, standard deviation.

Almost all patients had MB-type leprosy (21 patients or about 91.3%), and most of them had been diagnosed with leprosy for more than 5 years. Table 2 shows that both groups had similar clinical characteristics.

**TABLE 2:** Clinical characteristics.

Clinical characteristics	MT group no. of patients (%)	GWI group no. of patients (%)	*P*-value
Total eyes	11 (47.8)	12 (52.2)	0.686
**Leprosy type**			
Paucibacillary	1 (4.3)	1 (4.3)	0.455
Multibacillary	10 (43.5)	11 (47.8)	
**Leprosy duration**			
<2 years	0 (0.0)	1 (4.3)	0.560
2–5 years	3 (13.0)	2 (8.7)	
>5 years	8 (34.8)	9 (39.1)	
**Leprosy treatment**			
Not finished yet	0 (0.0)	0 (0.0)	N/A
Finished	11 (47.8)	12 (52.2)	
Unfinished	0 (0.0)	0 (0.0)	
**Lagophthalmos distance in central area preoperative (mm)**			
Without pressure	5.42 ± 2.54	4.82 ± 2.40	0.873
Gentle pressure	3.21 ± 1.99	3.09 ± 2.91	0.925

### Treatment effectivity and efficiency

Lagophthalmos distances at the central area measured with gentle pressure decreased significantly (*p* < 0.05) after the surgery compared with the preoperative result until the 3-month observation period both within the MT and GWI groups. In the MT group, the lagophthalmos distance with pressure significantly decreased until the 3-month observation at the nasal area (*p* < 0.05), and until the 1-month observation at the temporal area (*p* < 0.05). In the GWI group, the lagophthalmos distance with pressure significantly decreased until 1-year observation at the nasal area (*p* < 0.05), and until 3-month observation at the temporal area (*p* < 0.05).

Lagophthalmos distances without pressure between the MT and GWI groups are shown in Table 3. Table 4 shows lagophthalmos distances with gentle pressure in both groups. The MT and GWI techniques showed no significant difference in decreasing lagophthalmos distances with or without gentle pressure at nasal, central, and temporal areas.

**TABLE 3:** Lagophthalmos distance without pressure (mm) in the MT and GWI groups.

Area	Follow up time	MT group				GWI group				*P*-value
		Mean/Median	SD/IQR	Δ	Range	Mean/Median	SD/IQR	Δ	Range	
Nasal	Pre	3.23	2.56		0–7	3.71	2.45		1–9	
	PostD1	0.36	0.67	2.87	0–2	1.29	2.07	2.42	0–5	0.724^a^
	PostD7	0.95	1.00	2.28	0–2.4	1.00	0.85	2.71	0–2	0.680^a^
	PostM1	1.55	1.83	1.68	0–6	0.75	0.97	2.96	0–3	0.264^a^
	PostM3	2.35	1.89	0.88	0–5.9	1.00	1.12	2.71	0–3	0.148^a^
	PostY1	1.71	1.50	1.52	0–4	1.00	1.00	2.71	0–2	0.168^a^
Central	Pre	4.82	2.40		1–8	5.42	2.54		1–10	
	PostD1	0.28	0.47	4.54	0–1	0.92	1.83	4.50	0–6	0.961^b^
	PostD7	0.84	1.02	3.98	0–3	1.00	1.21	4.42	0–3	0.617^b^
	PostM1	1.97	3.08	2.85	0–9	1.08	2.07	4.34	0–7	0.139^b^
	PostM3	2.99	2.85	1.83	0–9	1.25	1.29	4.17	0–3	0.044^b^
	PostY1	1.43	1.27	3.39	0–3	1.64	1.84	3.78	0–5	0.383^b^
Temporal	Pre	2.91	2.81		0–10	2.50	1.83		0–5	
	PostD1	0.00	0.00	2.91	0–0	0.46	0.9	2.04	0–3	0.487^a^
	PostD7	0.28	0.47	2.63	0–1	0.28	0.47	2.63	0–1	0.928^a^
	PostM1	0.68	1.35	2.23	0–4	0.68	1.35	2.23	0–4	0.833^a^
	PostM3	1.06	1.63	1.85	0–4.7	1.06	1.63	1.85	0–4.7	0.740^a^
	PostY1	0.57	0.79	2.34	0–2	0.57	0.79	2.34	0–2	0.805^b^

SD, standard deviation; Δ, delta; Pre, preoperative; PostD1, postoperative day 1; PostD7, postoperative day 7; PostM1, postoperative month 1; PostM3, postoperative month 3; PostY1, postoperative year 1; ^a^independent *t*-test; ^b^Mann–Whitney test.

**TABLE 4:** Lagophthalmos distance with gentle pressure (mm) in the MT and GWI groups.

Area	Follow up time	MT group				GWI group				*P*-value
		Mean/Median	SD/IQR	Δ	Range	Mean/Median	SD/IQR	Δ	Range	
Nasal	Pre	2.18	2.28		0–8	2.29	1.54		0–4.5	
	PostD1	0.13	0.32	2.05	0–1	0.66	1.61	1.63	0–5	0.671^a^
	PostD7	0.39	0.76	1.79	0–2	0.29	0.62	2.00	0–2	0.203^b^
	PostM1	0.41	1.20	1.77	0–4	0.50	0.67	1.79	0–2	0.334^a^
	PostM3	1.06	1.96	1.12	0–5	0.58	0.66	1.71	0–2	0.520^a^
	PostY1	1.00	0.89	1.18	0–2	0.42	0.78	1.87	0–2	0.085^a^
Central	Pre	3.09	2.91		0–9	3.21	1.99		0–6	
	PostD1	0.00	0.00	3.36	0–3	0.83	1.94	2.38	0–5	0.547^a^
	PostD7	0.28	0.90	3.14	0	0.75	1.48	2.46	0–5	0.736^a^
	PostM1	1.04	2.12	2.07	0–6	0.42	0.90	2.79	0–3	0.316^b^
	PostM3	1.33	2.01	3.14	0–5	0.75	0.86	2.46	0–2	0.382^a^
	PostY1	0.43	1.27	1.57	0–3	0.83	1.84	2.38	0–5	0.238^a^
Temporal	Pre	2.04	2.49		0–8	1.29	1.30		0–3	
	PostD1	0.18	0.60	1.86	0–2	0.00	0.00	1.29	0–0	0.976^b^
	PostD7	0.09	0.30	1.95	0–1	0.83	0.28	0.46	0–1	0.786^b^
	PostM1	0.41	0.97	1.63	0–3	0.83	0.28	0.46	0–1	0.833^b^
	PostM3	0.72	1.27	1.32	0–4	0.16	0.39	1.13	0–1	0.786^b^
	PostY1	0.78	0.78	1.26	0–2	0.85	1.07	0.44	0–3	0.535^b^

SD, standard deviation; Δ,delta; Pre, preoperative; PostD1, postoperative day 1; PostD7, postoperative day 7; PostM1, postoperative month 1; PostM3, postoperative month 3; PostY1, postoperative year 1; ^a^independent *t*-test; ^b^Mann–Whitney test.

This study took account of confounding variables such as age, gender, level of education, leprosy type, and duration of leprosy. In the multivariate analysis, these variables significantly affected the lagophthalmos distance without pressure. Meanwhile, the lagophthalmos distance with gentle pressure was significantly affected by age and level of education.

The TBUT improved significantly (*p* < 0.05) in the GWI group after 1-year observation; meanwhile, in the MT group, no significant improvement was found in TBUT during the observation. In the GWI group, the Schirmer test without anesthesia only showed significant improvement at 1-day observation, and the Schirmer test with anesthesia had significant improvement at 1-day observation as well as at 3-month observation (*p* < 0.05). In the MT group, no significant improvement was found in the Schirmer test without anesthesia, but the Schirmer test with anesthesia showed significant improvement after 1-year observation (*p* < 0.05) (Table 5).

**TABLE 5:** Subjective and objective tear assessments using OSDI (total score), TBUT (seconds), and Schirmer tests (millimeter) of patients in the MT and GWI groups.

Test	Follow up time	MT group			GWI group			*P*-value
		Mean/Median	SD/IQR	Δ	Mean/Median	SD/IQR	Δ	
OSDI	Pre	29.74	5.83		31.33	11.11		
	PostD1	19.45	9.27	-10.28	21.33	12.62	-10	0.941^a^
	PostD7	14.00	6.58	-15.74	14.83	8.77	-16.5	0.809^a^
	PostM1	11.27	8.94	-18.47	16.17	9.48	-15.16	0.400^a^
	PostM3	8.09	7.94	-21.65	9.75	7.43	-21.58	0.880^b^
	PostY1	11.50	8.58	-18.24	15.09	12.44	-16.24	0.226^a^
TBUT	Pre	4.20	3.04		2.10	0.73		
	PostD1	5.0	2.98	0.80	3.09	1.86	0.99	0.566^b^
	PostD7	4.0	3.09	-0.20	2.41	1.56	0.3‘	0.614^a^
	PostM1	3.81	1.47	-0.39	3.08	1.78	0.98	0.258^a^
	PostM3	5.10	3.24	0.90	4.0	1.78	1.9	0.494^a^
Schirmer without anesthesia	Pre	20.45	9.06		12.5	9.66		
	PostD1	24.36	9.32	3.91	20.18	10.13	7.68	0.685^a^
	PostD7	19.82	10.08	-0.63	13.16	9.23	0.66	0.771^a^
	PostM1	20.27	8.92	-0.18	15.58	10.06	3.08	0.495^a^
	PostM3	12.10	10.71	-8.35	13.18	11.66	0.68	0.127^a^
	PostY1	19.66	7.76	-0.79	17.14	11.67	4.64	0.564^a^
Schirmer with anesthesia	Pre	16.64	9.68		14.25	10.64		
	PostD1	21.09	10.68	4.45	22.16	10.93	7.91	0.539^a^
	PostD7	21.36	9.31	4.72	18.66	6.44	4.41	0.943^a^
	PostM1	19.54	9.34	2.90	18.58	8.67	4.33	0.760^a^
	PostM3	19.40	10.52	2.76	18.36	7.00	4.11	0.788^a^
	PostY1	25.33	11.36	8.69	18.28	10.30	4.03	0.141^a^

SD, standard deviation; Δ, delta; Pre, preoperative; PostD1, postoperative day 1; PostD7, postoperative day 7; PostM1, postoperative month 1; PostM3, postoperative month 3; PostY1, postoperative year 1; ^a^independent *t*-test; ^b^Mann–Whitney test.

Epitheliopathy was observed pre- and post-surgery. Epitheliopathy improvement occurred in 13 of 23 eyes, 6 of which (54.55%) were in the MT group and 7 (58.33%) were in the GWI group, with no significant difference between both groups. No epitheliopathy was found at pre- and post-surgeries in one eye (4.3%), whereas nine eyes (39.1%) experienced this condition with no improvement after a 3-month observation period (Table 6).

**TABLE 6:** Epitheliopathy improvement between the MT and GWI groups.

Follow up time	MT group *N* = 11	GWI group *N* = 12	*P*-value
Post D1	0 (0.00%)	3 (25.00%)	0.124^a^
Post D7	3 (27.27%)	4 (33.33%)	0.556^a^
Post M1	5 (45.45%)	5 (41.67%)	0.593^a^
Post M3	6 (54.55%)	7 (58.33%)	0.593^a^

PostD1, postoperative day 1; PostD7, postoperative day 7; PostM1, postoperative month 1; PostM3, postoperative month 3; ^a^chi-square test.

Corneal exposure was observed to assess part of the cornea exposed while the eyes were closed. Table 7 shows that the corneal exposure degree at pre- and post-surgery follow-up appointments in the MT group and the GWI group indicates no significant difference between both groups. The results showed improvement after surgery on the first and seventh days in both groups, but the degree of exposure increased in the first month in the MT group and in the third month in the GWI group.

**TABLE 7:** Corneal exposure degree (mm) between the MT and GWI groups.

Follow up time	MT group			GWI group			*P*-value
	Mean	SD	Δ	Mean	SD	Δ	
Pre	0.50	0.24		0.33	0.18		
Post D1	0.00	N/A	0.50	0.00	N/A	0.33	0.873
Post D7	0.00	N/A	0.50	0.00	N/A	0.33	0.873
Post M1	0.18	0.12	0.32	0.00	N/A	0.33	0.873
Post M3	0.27	0.14	0.23	0.08	0.08	0.22	0.866

SD, standard deviation; Δ, delta; Pre, preoperative; PostD1, postoperative day 1; PostD7, postoperative day 7; PostM1, postoperative month 1; PostM3, postoperative month 3; Mann–Whitney test.

Table 8 shows corneal sensitivity pre- and post-surgery in all four quadrants between the MT and GWI groups. Corneal sensitivity improvement in both groups was insignificantly different.

**TABLE 8:** Corneal sensitivity between the MT and GWI groups.

Corneal quadrant	Follow up time	MT group			GWI group			*P*-value
		Median	Range	Δ	Median	Range	Δ	
Superior	Pre	60	(45–60)		60	(40–60)		
	PostM3	60	(40–60)	0.00	60	(40–60)	0.00	0.562
Nasal	Pre	60	(40–60)		60	(35–60)		
	PostM3	60	(40–60)	0.00	60	(45–60)	0.00	0.754
Temporal	Pre	60	(45–60)		60	(50–60)		
	PostM3	60	(45–60)	0.00	60	(50–60)	0.00	0.377
Inferior	Pre	60	(50–60)		60	(50–60)		
	PostM3	60	(40–60)	0.00	60	(25–60)	0.00	0.295

SD, standard deviation; Δ, delta; Pre, preoperative; PostM3, postoperative month 3; Mann–Whitney test.

Surgical technique safety, duration of surgery, and cost of surgery were observed to determine procedure efficiency. In addition, the complication was evaluated from the first day to 12 months post-surgery to rate surgical technique safety. The observation result showed that there was no complication found in the MT group, while two eyes (15%) in the GWI group experienced implant extrusion. Furthermore, the surgery duration for those in the MT group (44,61 ± 11,29 min) was insignificantly different from those in the GWI group (43,81 ± 15,03 min). In terms of cost, the mean cost of surgery in the MT group was 1.488.357, 14 ± 18.156,82 IDR, while that in the GWI group was 1.488.357, 14 ± 18.156,82 IDR. Only two patients who had implant extrusion underwent the MT technique as a reparative procedure. The final mean cost of those in the GWI group after complication correction was significantly higher (3.017.437,54 ± 560.823,97 IDR) than of those in the MT group (*p* < 0.05).

## Discussion

In this study, in general, lagophthalmos is found in patients with MB-type leprosy. According to a previous study, patients with 100% leprosy who experienced ocular complications were diagnosed with the MB type (borderline and lepromatous leprosy types) (5). This was related to the ability of *Mycobacterium leprae* to invade the Schwann cells at facial cranial nerve (CN) VII endings. These peripheral nerves innervate the orbicularis muscles of the eyelid.

This study showed a decrease in the lagophthalmos distance after the surgery without significant differences between the MT and GWI groups. Modified tarsorrhaphy was performed with a combination of three procedures: levator recess, lateral tarsorrhaphy of 10 mm, and lateral canthopexy/lateral tarsal strip (LTS). This was performed at the temporal side due to its large area; therefore, patients’ vision field would not be limited. Tarsorrhaphy was carried out at about 10 mm from the lateral canthus to provide an adequate visual function, and this technique yielded more aesthetically pleasing and more comfortable results. Moreover, it would not disrupt the tear flow into the medial punctum. Another study reported a permanent lateral tarsorrhaphy procedure in four lagophthalmos eyes (2–3.5 mm) with the lower lid retracted laterally and the upper lid retracted medially. This technique resulted in a smaller palpebral width and limited vision field (6). A significant difference in the lagophthalmos distance without pressure was only found in the third-month follow-up (*p* = 0.044). In the MT group, no weight was placed on the eyelids, and after 3 months of the wound-healing process, the swelling of the eyelid reduced, and there was a significant difference in the lagophthalmos distance compared with the GWI group.

The ideal GWI weight could be calculated preoperatively by putting trial weight on the pretarsal lid from 0.6 g and adding 0.2 g gradually until the lagophthalmos is reduced by 50% without inducing ptosis more than 2 mm (7). Another study suggested adding 0.2 g from the ideal weight to obtain better eyelid closure (8). In this study, the GWI group preoperative mean eyelid margin distance was 5.38 ± 2.43 mm. To make the implant easier to obtain, the researchers used 1.5-g gold weight for all patients as the average load as opposed to implants with a different ideal weight for each eye. In the GWI group, the lagophthalmos distance decreased significantly with a more stable result. The patients in this group experienced limited vision field when the eyes were open due to the weight in their upper lids.

A study on dry eyes in patients with leprosy is currently developing. This dry eye condition in patients with leprosy is related to several mechanisms, such as decreasing tear component secretion and increasing evaporation. Subjective dry eye symptoms were evaluated through OSDI questionnaires. The OSDI score showed significant improvement from pre- to post-surgery within each group, with no significant difference between them. A decrease in the lagophthalmos distance leads to reduced evaporation and a more comfortable experience after undergoing the surgery, as indicated by the OSDI score (9).

In addition, lagophthalmos could cause conjunctival goblet cell impairment, which leads to a decrease in mucin production. A study showed that human goblet cells did not have nerve endings, but their secretion could be activated with sympathetic and parasympathetic nerve stimulation (10). Patients with leprosy had a high prevalence of Meibomian gland dysfunction due to *Mycobacterium leprae* infiltration and infection. Meibomian gland dysfunction could lead to gland atrophy, increasing tear osmolarity and reducing defense of the anterior surface of the cornea (11). *Mycobacterium leprae* could also directly invade lacrimal glands, causing a reduction in aqueous component production (12). Objectively, tear film stability was evaluated by using the TBUT, and the Schirmer test with and without anesthesia. The test results showed that the tear film stability was less than normal, in both groups at preoperative assessment, but this increased insignificantly post-surgery. A study also showed that the TBUT in patients with MB-type leprosy was significantly lower than that in normal eyes (13). Schirmer tests pre- and post-surgery were still in the normal range, suggesting a normal function of the lacrimal gland in this study. This result was similar to that of a previous study where there was no significant difference in the Schirmer test results between the patients with MB- or PB-type leprosy and the control group comprising normal eyes (13).

A previous study suggested that male individuals have a greater lower eyelid pressure, which results in relatively a slow healing of epitheliopathy (14). The weight of the implant put pressure on the upper eyelid, which was likely to cause less epitheliopathy improvement for those in the GWI group.

The corneal exposure improvement in the GWI group was due to the gold implant placed on the upper eyelid, which is similar to a previous study that showed that the corneal function remained constant in 99.5% of the patients in the 2-year evaluation (15). Meanwhile, in the MT group, the lagophthalmos distance increased after the healing process was complete.

A decrease in corneal sensitivity in patients with leprosy is caused by *Mycobacterium leprae* invasion to the maxillary branch of CN V, which innervates the corneal surface. Corneal sensitivity decreases over time as, in general, it is found in patients with MB-type leprosy and causes nerve atrophy, sensory function disruption, and lagophthalmos, which lead to corneal xerosis and tear film instability, and also indirectly causes corneal microtrauma (16). According to the systematic review of seven studies, impaired corneal sensitivity incidence varied between 8.1 and 59.2%, depending on disease duration and anterior segment abnormalities (17).

Postoperative complications were found in two patients (15%) in the GWI group after 1-year observation, whereas no complication was found in MT group patients. A previous study reported 50% of patients experienced extrusion after 1-year observation, from 3 to 12 months (4). However, in this study, a different GWI technique was used to prevent extrusion as the implant was placed above the tarsal lid under the aponeurosis levator muscle. Those two patients in the GWI group with implant extrusion underwent the MT procedure as repair surgery, and they felt satisfied with the results. One of the patients who belonged to the GWI group expressed discomfort with the implant after the 3-month evaluation. The patient experienced a very limited vision field, which interfered with his job as a motorbike driver, and requested to receive the MT surgery. Therefore, the MT technique to replace the previous GWI technique was performed on the patient’s eyes, and the patient felt more comfortable after the second surgery. Another common complication in the GWI group was allergic reactions. Gold has an inert nature, but it can trigger allergic reactions in some patients. Therefore, an allergy examination is necessary, particularly for patients with a previous history of gold or metal allergy.

The duration of surgery was insignificantly different for both groups. Even though the MT technique consisted of a three-step procedure, more procedures than those used in the GWI technique, both techniques had the same level of difficulty when suturing the implant to the tarsal plate. The GWI technique had to be performed carefully to avoid the pretarsal orbicularis muscle tear as the implant was placed above the tarsal plate, underneath the levator aponeurosis. Another consideration is the cost of surgery; the cost was significantly higher in the GWI group since gold as the implant material, which is a higher value, was used in the GWI surgery, even though the surgery preparation cost (laboratory examinations, radiology imaging) of both MT and GWI procedures were similar. Moreover, for two patients who experienced implant extrusion and underwent an additional MT procedure, the total cost of surgery in the GWI group was higher. The surgery cost is important to be considered when deciding on lagophthalmos treatment in patients with leprosy since the patients in most cases are from a low socioeconomic class. Therefore, the MT technique is recommended as an alternative treatment for paralytic lagophthalmos in patients with leprosy as this technique was as effective as the GWI technique but more efficient than the GWI technique. The MT group showed no complication and felt more satisfied with the results than those in the GWI group.

## Conclusion

The effectiveness of the MT technique was similar to that of the GWI technique in decreasing the eyelid margin distance (lagophthalmos distance) with and without gentle pressure, ameliorating subjective and objective tear assessments, improving epitheliopathy occurrence, decreasing corneal exposure, and improving corneal sensitivity. However, the MT technique was more efficient, had no complication, and had a lower cost of surgery than the GWI technique. The duration of surgery for both techniques was similar. Modified tarsorrhaphy can be considered appropriate for paralytic lagophthalmos surgical treatment in patients with leprosy.

### Limitation

Lagophthalmos in patients with leprosy typically occurs over a long period of time, which allows them to adjust. Although patients with lagophthalmos are numerous, it might be challenging to locate patients who are willing to undergo surgery at community-based health institutions. Moreover, this study was held during the COVID-19 pandemic. Consequently, the number of samples was limited.

## Data availability statement

The original contributions presented in this study are included in the article/Supplementary material, further inquiries can be directed to the corresponding author.

## Ethics statement

The studies involving human participants were reviewed and approved by Medical and Health Research Ethics Committee (MHREC) Faculty of Medicine, Public Health and Nursing Universitas Gadjah Mada – Dr. Sardjito General Hospital, Yogyakarta, Indonesia. The patients/participants provided their written informed consent to participate in this study.

## Author contributions

YI designed the concept, planned the study, and led the project administrations. YI and MN obtained the data collection and performed the data analysis. YI, MN, TG, ID, and HS were involved in data interpretation and validation. All authors read and approved the final manuscript.
