# Supplementary material for: Modified tarsorrhaphy versus gold weight implant technique for paralytic lagophthalmos treatment in patients with leprosy: One-year observation of a randomized controlled trial study
Source: Front Med (Lausanne). 2023 Jan 4;9:941082. doi: 10.3389/fmed.2022.941082 (PMC9845573; doi:10.3389/fmed.2022.941082)
Supplement: Supplementary file 1 [file Data_Sheet_1.pdf]

RESEARCH PROTOCOL

Title	Effectivity and Efficiency Comparison between Modified Tarsorrhaphy and Gold Weight Implant Technique as Operative Management of Paralytic Lagophthalmos in Leprosy Patients
Reference Number	KE/0335/03/2018
Principal Investigator	Yunia Irawati, MD
Investigator Affiliation	Plastic Reconstructive Surgery Division, Ophthalmology Department, Faculty of Medicine, University of Indonesia, dr. Cipto Mangunkusumo National Hospital, Jakarta, Indonesia
Address	Jl. Kimia No. 8-10 Menteng Jakarta Pusat
Phone Number	+62816789595
Funding	Researchers received no sponsor involved in funding

1. INTRODUCTION

a. Background

Leprosy is a chronic infection that caused by *Mycobacterium leprae (M. leprae)* which attacked skin, peripheral nerve, upper airway mucosa, annd eye. *M. leprae* is an obligate intracellular acid-fast bacillus (AFB), rod-shaped, and multiply inside histiocytes, Schwann cells, muscle cells, and vascular endothelia. Leprosy can be transmitted through skin-to-skin direct contact in the long term. Factors that influence the spread of leprosy are: *M. leprae* pathogenesis, mode of transmission, imunology status, individual genetics, socio-economy status and environmental factor (Wisnu, *et al.* 2016; Fitness, Tosh & Hill 2002).

According to World Health Organization (WHO), leprosy can be divided into 2 types, paucibacillary (PB) and multibacillary (MB). Ridley and Jopling classified leprosy into several zones of a spectrum, which are TT (polar tuberculoid), BT (borderlinve tuberculoid), BB (mid-borderline), BL (boderline lepromatous), LL (polar lepromatous). MB type includes LL, BL, and BB types, whereas PB type include TT and BT types (Wisnu, *et al.* 2016)

Leprosy prevalece universally in the end of 2014 is 175.554 and the number of new cases is 213.899 (WHO 2016b; Kemenkes 2016b; WHO 2016a). Leprosy endemically found in developing countries in the tropic and subtropic areas, such as Asia, Africa, and Latin America because limited access to health facility, education, and lower social and economic

welfare. Southeast Asia is a developing countries region with the highest number of new leprosy cases in the world of 154.834 cases in 2014. Indonesia is one of the developing countries with highest new leprosy case amount in Southeast Asia and the third highest in the world after India and Brazil (Hogeweg 2010; WHO 2016a). In 2015, there was 17.202 new leprosy case in Indonesia with 84.5% of it are MB typed. Until recently, leprosy is still one of the health problems in Indonesia. Even though leprosy has been eliminated in 2000, leprosy cases in Indonesia is still relatively static (Kemenkes 2016b).

Leprosy is a systemic infection with ocular complication leading to visual impairment that could end in blindness (Rahinam 2010). Most ocular complication was found in elderly patient with MB type leprosy (Hogeweg 2010; Courtright & Lewallen 2006; Lewallen & Courtright 2012). About 70-75% of leprosy patients in the world experienced ocular complication, with 10-50% in severe condition and 5% end in blindness (Grzybowsi, et al 2015). Blindness can be caused by several factors, such as leprosy type, duration, medication effect, and eye disorder management. There was not much data available regarding leprosy, although a survey estimated 25-50% of the population of leprosy patients or who have recovered from leprosy have eye disorders (Courtright et al 2002). WHO divided eye disorders in leprosy into 2 grades, according to visual acuity (VA): grade 1 with VA $\geq$ 6/60 and grade 2 with VA < 6/60 (Ebenezer 2016).

Courtright et al (2002) in India, Phillipines, and Ethiopia found that 11% of MT type leprosy patients have eye disorders which potentially lead to blindness, and 2,8% of them will be blind (Courtright et al. 2002). Study done by Singh et al (2014) showed eye disorders were found in 39,4% leprosy patients with 13,07% grade 1 visual impairment and 19,86% grade 2 visual impairmennt (Singh et al. 2014). Another study by Rao (2015) showed the most common eye disorders are madarosis (58,33%), corneal involvement (38,8%), lagophthalmos (28,89%), while blindness found in 30,55% patients (Rao 2015).

Data regarding eye involvement in leprosy patients in Indonesia have not been published much. A survey in rural area of Gresik and Lamongan, East Java, in 1997-1998 showed that there were eye disorders in 7% out of 112 leprosy patients with disability (Schreuder et al 2002). A study done in Cirebon in 2010-2011 showed that grade 2 visual impairment were found in 5,6% of the new leprosy cases (Peters et al. 2013). Another study by Arifin R from March 2016 to May 2017 in Tangerang showed from 138 MB type leprosy patients, 25 patients had uveitis and 11 patients of them (44%) had bilateral uveitis. In this study, uveitis found mostly in men (80%), MB type LL leprosy (64%), age 60 years old, and duration of leprosy 42 years (Arifin 2017).

Clinical manifestations of eye disorders in leprosy were dividied into adnexal/ extraocular and intraocular manifestations. Eye disorders pathogenesis in leprosy divided into tuberculoid (TT) and lepromatous (LL) types. Eye disorder in TT type leprosy mostly happened in adnexal area, including eyelid, mainly if there was a focal lesion above facial cranial nerve (CN VII) that innervates orbicularis oculi muscle. This process usually happened unilaterally and rarely leading to intraocular manifestations. LL type process happened more slowly, more symmetric than TT type and more commonly leads to intraocular manifestations. LL type patients have higher risk in ocular complications (Grzybowsi, et al. 2015; Ebenezer 2016; Sjamsoe & Sjamsoe-Daili 2003).

Oribicularis oculi muscle works to close eyelid and innervated by CN VII. Lagophthalmos and disturbance in tear excretion could happened due to nerve paralysis. Lagophthalmos is a condition where eyelid cannot close completely. If lagophthalmos was not soon detected and corrected, it could lead to corneal opacity and threat visual acuity (Grzybowsi et al. 2015; Hogeweg 2001; Parikh et al. 2009; Lewallen et al. 2000; Lewallen & Courtright 2012). Leprosy patients with lagophthalmos usually have lesion somewhere in the facial area, and usually have disability also in their arms and legs when diagnosed with leprosy, compared to patients without lagophthalmos (Courtright et al 2002; rathinam 2010; Lemallen & Courtright 2012). About 2-4% leprosy patients have lagophthalmos before diagnosed or during MDT (multi drug therapy) treatment, 6-10% were elderly patients and had disability (Hogeweg 2010). According to data in 2013-2015 in Dermatovenereology Policlinic dr. Cipto Mangunkusumo Hospital, lagophthalmos was found in 2,4% of 501 new leprosy cases. All patients with lagophthalmos were MT type leprosy patients (Lasrindy 2016).

Lagophthalmos pathogenesis started when *M. leprae* invade peripheral nerve (Schwann cell) where there were protein bridging to Schwann cell cytoskeleton, which are laminin-2, α-dystroglycan, β-dystroglican, and dystrophin. *M. leprae* would enter Schwann cell endoneurium, and then stimulate inflammation response in nerve cells. Invaded nerve cells are facial cranial nerve (CN VII) zygomatic and temporal branches, which leads to orbicularis oculi muscle paralysis so that eyelid could not close completely (paralytic lagophthalmos), also ophthalmic branch of trigeminal cranial nerve (CN V) which leads to decrease in corneal sensibility. Paralytic lagophthalmos with decreased corneal sensibility could cause corneal opacity and decreased visual acuity that leads to blindness (Lewallen & Courtright 2012; Sjamsoe & Sjamsoe-Daili 2003; Gryzbowsi et al 2015; Ebenezer 2016).

Corneal opacity due to lagophthalmos was the most common cause of blindness in leprosy (Lewallen et al. 2000).

Lagophthalmos management depends on duration and size of lagophthalmos, also the presence of corneal exposure. In lagophthalmos with eyelid gap >6 mm, presence of corneal exposure, and duration >6 months, the ideal treatment is with eyelid reconstructionn surgery (Hogeweg 2010). There were several reconstruction surgery techniques for lagophthalmos, although these techniques gave various results. The easiest surgery technique to manage lagophthalmos is permanent lateral tarsorrhaphy, which was done with joining upper and lower lateral side of the eyelid so that patient could still see from the central side. Even though, this technique has its limitation, which is limited temporal visual field and poor cosmetic result (Hogeweg 2001; Hogeweg 2010; Rajak et al. 2015).

Therefore, this study attempts to combine tarsorrhaphy technique with another eyelid reconstruction techniques, which are levator recess and canthopexy. Levator recess technique aims to weaken levator muscle to enable eyelid closure passively, while canthopexy aims to tighten lower eyelid, because in lagophthalmos due to orbicularis paralysis, there were tendency of lower lid laxity.

Another common technique to reconstruct CN VII paralysis as lagophthalmos management is upper eyelid loading, using gold weight implant (Rahman & Sadiq 2007). Gold weight implant has a high success rate as lagophthalmos treatment. This technique uses gold implant and attach it into the upper eyelid so that the weight of the gold pulled by the gravity and the eyelid closes passively when palpebral levator muscle relaxed. The advantages of this technique are: increasing eyelid closure with gravity pull, protecting cornea from exposure, and good cosmetic result. The disadvantages of gold weight implant technique are: expensive material, risk of complications, such as inflammation, allergic reaction, extrusion, migration, and astigmatism (Bladen et al 2012; Chi 2016). Wagh et al (2016) showed gold weight implant as the most effective treatment for paralytic lagophthalmos from keratopathy improvement and decrease in ocular surface defects.

Generally, eye disorders in leprosy patients were poorly diagnosed in primary health facility. Ocular manifestations in leprosy were assessed by general physician, dermatovenereology specialist, and ophthalmologist with different level of knowledge so that ocular complication in leprosy may still be underdiagnosed (Ebenezer 2016). Every eye disorders in leprosy patients, including lagophthalmos, need early detection and early treatment to prevent blindness. Eyelid reconstruction surgery is needed to correct paralytic lagophthalmos in leprosy patients, but not all health facility has a trained ophthalmologist to

do eyelid reconstruction surgery. Limited access and cost for treatment in tertiary health facility, leprosy patients commonly came when ocular complications have reached severe visual impairment. Therefore, this study is useful in ophthalmology field for several reasons:

1. There were no studies comparing effectivity and efficiency of various reconstruction surgery techniques for lagophthalmos in leprosy patients.
2. There were no complete data in Indonesia regarding incidence and prevalence of lagophthalmos due to leprosy.
3. With this research, better leprosy management can be carried out to prevent blindness from lagophthalmos in leprosy patients.

b. Objectives

- Main objective

Comparing effectivity and efficiency between modified tarsorrhaphy and gold weight implant technique as operative treatment for paralytic lagophthalmos in leprosy patients

- Specific objectives

Assessing the success of modified tarsorrhaphy and gold weight implant techniques as reconstructive treatment for lagophthalmos in leprosy patients according to:

a. Lagophthalmos distance pre- and post-operative
b. Dry eyes assessments (subjective assessment with OSDI questionnaire and objective assessment with TBUT, Schirmer test without anesthesia and Schirmer test with anesthesia)
c. Epitheliopathy
d. Corneal exposure
e. Corneal sensibility
f. Complication intra-operative and post-operative, which are bleeding, infection, granulation, implant extrusion
g. Duration of surgery
h. Cost of surgery

c. Hypothesis

Modified tarsorrhaphy is more effective and efficient than gold weight implant technique as operative treatment for paralytic lagophthalmos in leprosy patients.

d. Trial Design

This study uses PROBE (Prospective Randomized Open-label Blinded-Endpoint) in which samples are randomized to allocate received intervention, principal investigator knows the intervention assigned according to randomization. Outcome measurements are carried out by three trained ophthalmologists as research team members, so that the measurements are done objectively and principal investigator is not biased.

2. METHODS

a. Study Setting

Target Population

Target population of this study are Paucibacillary (PB) or Multibacillary (MB) type leprosy patients with unilateral or bilateral lagophthalmos in Indonesia.

Study Population

Study population of this study are PB or MB type leprosy patients with unilateral or bilateral lagophthalmos who have completed MDT therapy in Jakarta, Makassar, or Tangerang.

Study Sample

Study population of this study are PB or MB type leprosy patients with unilateral or bilateral lagophthalmos who meet inclusion and exclusion criteria at Jakarta Eye Center Hospital (Jakarta), Dr. Tadjudidin Chalid Hospital (Makassar), or dr. Cipto Mangunkusumo Kirana Eye Hospital (Jakarta).

b. Eligibility Criteria

Inclusion Criteria

1. PB or MB type leprosy patients with unilateral or bilateral lagophthalmos who have not done eyelid reconstruction surgery before
2. Age of 18 years old or older and able to have surgery with local anesthesia
3. Willing to take part as clinical trial subject and sign an informed consent

4.

Exclusion Criteria

1. Refuse to sign informed consent
2. History of eyelid reconstruction surgery
3. Patient with leprosy in acute stage (< 6 months) or in steroid therapy
4. Eyelid laxity > 8 mm

Drop Out Criteria

1. Patient who do not come for follow up appointments as assigned by research team members
2. Patient who withdrew during the study

c. Interventions

Modified Tarsorrhaphy Technique

1. Patient preparation and aseptic procedures on subject eye
2. Marking at eyelid crease skin
3. Subcutaneous anesthesia using Lidocain HCl 20 mg and Epinephrine 0.0125 mg/ml (Pehacain(R)) on upper eyelid and lateral side of the eye
4. Incision at skin crease and dissection of orbicularis muscle to clean superior tarsal border
5. Levator recess procedure with local anesthesia injection at conjunctiva to separate levator aponeurosis from conjunctiva. Open orbital septum at the level of superior tarsal. Excision of levator aponeurosis at its insertion site in the tarsal. Separate aponeurosis levator from conjunctiva as far as possible
6. Do canthotomy and lateral cantholysis. Excision of upper and lower eyelid margin as long as 10 mm from lateral canthal to central. Lateral tarsorrhaphy suture using polyglactin 6-0 with reverse cutting needle
7. Do canthopexy/lateral tarsal strip (LTS) using polyglactin 5-0 with reverse cutting needle and canthoplasty using polyglactin 6-0 with reverse cutting needle
8. Suture skin to form lid crease (skin-tarsal-skin) at 3 points and suture skin in between using polypropilene 6-0 with reverse cutting needle.

Gold Weight Implant Technique

1. Patient preparation and aseptic procedures on subject eye
2. Marking at eyelid crease skin
3. Subcutaneous anesthesia using Lidocain HCl 20 mg and Epinephrine 0.0125 mg/ml (Pehacain^(R)) on upper eyelid and lateral side of the eye
4. Incision at skin crease and dissection of orbicularis muscle to clean superior tarsal border
5. Open orbital septum at middle border of superior tarsal
6. Eversion of upper eyelid, do subconjunctiva anesthesia to separate aponeurosis from conjunctiva
7. Levator recess procedure with excision of levator aponeurosis at its insertion site in the tarsal. Separate aponeurosis levator from conjunctiva
8. Implant placed at superior tarsal border underneath levator recess with attachment to septal frill, and then fixed at tarsal using polypropilene 6-0 with reverse cutting needle.
9. Suture orbicularis muscle to cover implant using polyglactin 6-0 with reverse cutting needle and suture skinn using polypropilene 6-0 with reverse cutting needle.

Intervention Monitoring

Researcher will monitor adverse events during and after surgery procedure. Any adverse events at any time during the study will be notified to the surgeon and will be treated by the surgeon or referred to related specialist.

Concomitant Care and Interventions

All subjects should have completed MDT (multi drug therapy) treatment for leprosy and not in steroid treatment. Subject with previous ophthalmic surgery were excluded from the study. Patients with surgical complication from this study will be treated and will still be observed in this study.

d. Outcomes

Effectivity Outcomes:

1. Lagophthalmos Distance

Lagophthalmos distance is measured at central (middle pupil), nasal (medial limbus), and temporal (lateral limbus) between upper and lower eyelid while eyelids are closed without pressure and with gentle pressure. Distances are measured using caliper/ruler in millimeters.

2. Dry Eyes Assessments

Dry eyes condition is assessed using several modalities, which are OSDI (Ocular Surface Disease Index) questionnaire, Tear Break-up Time (TBUT), Schirmer Test with anesthesia and without anesthesia.

2.1. Ocular Surface Disease Index (OSDI) Questionnaire

Subjective symptoms of dry eyes are assessed using OSDI questionnaire that consists of 12 questions regarding dry eyes symptoms. Each question answered with 0-4 Likert scale (0: never, 1: seldom, 2: sometimes, 3: often, 4: always). Final score is calculated with: (total score x 100)/(total questions answered x 4). Final score then categorized into: normal (0-12), mild dry eye (13-22), moderate dry eye (23-32), or severe dry eye (33-100).

2.2. Tear Break-up Time (TBUT)

TBUT is measured with putting a fluorescent paper strip on bulbar conjunctiva and ask subject to blink. Then examine eye using cobalt blue light with a slit lamp, while subject holds eye open. The duration between eyelid opens and appearance of dry spot on corneal surface is the tear film break-up time, and measured in seconds. TBUT less than 10 seconds shows tear film instability.

2.3. Schirmer Test

Schirmer test aims to assess tear film secretion. This test using Whatman filter paper that put in lower lid fornix at third lateral side, and subject closes eyes for 5 minutes. Wet part of the filter paper then measured in millimeters to determine the result. Schirmer test is done in two ways: without

anesthesia and with anesthesia before putting the paper in the fornix. In normal eyes, wet part of the Whatman paper reaches 10 millimeters after 5 minutes.

3. Epitheliopathy

Epitheliopathy is examined with fluorescent staining with cobalt blue light using slit lamp. Epitheliopathy is measured with the presence of epitheliopathy or no epitheliopathy.

4. Corneal Sensibility

Corneal sensibility is examined with esthesiometer in millimeters. Esthesiometer has a filament as long as 60 mm, the tip of the filament is softly touched to the cornea and blinking reflex is observed. Corneal sensibility is categorized into: normal (55-60 mm), mild hypoesthesia (50-54 mm), hypoesthesia (40-49 mm), severe hypoesthesia (30-39 mm), and very severe hypoesthesia (<30 mm).

5. Corneal Exposure

Corneal exposure is exposed corneal distance when eyes closed in millimeters.

Efficiency Outcomes:

1. Safety of Surgery

Safety of surgery will be assessed with incidence of complications during and post-operative (bleeding, infection, granulation, implant extrusion, etc.).

2. Duration of Surgery

Duration of surgery in minutes from first incision until the procedure is done.

3. Cost of Surgery

Cost of tools and materials used for the surgery, laboratory examinations, and medications.

e. Participant Timeline

<u>Time Schedule</u>

Lagophthalmos distance and dry eyes assessments (OSDI questionnaire, TBUT, Schirmer test without anesthesia, Schirmer test with anesthesia), epitheliopathy, corneal sensibility, and corneal exposure will be assessed at 1 day, 7 days, 1 month, 3 months, and 1 year post-operatively. Complication and cost of surgery will be assessed intra-operative and post-operative until 1 year. Duration of surgery will be assessed during the surgery.

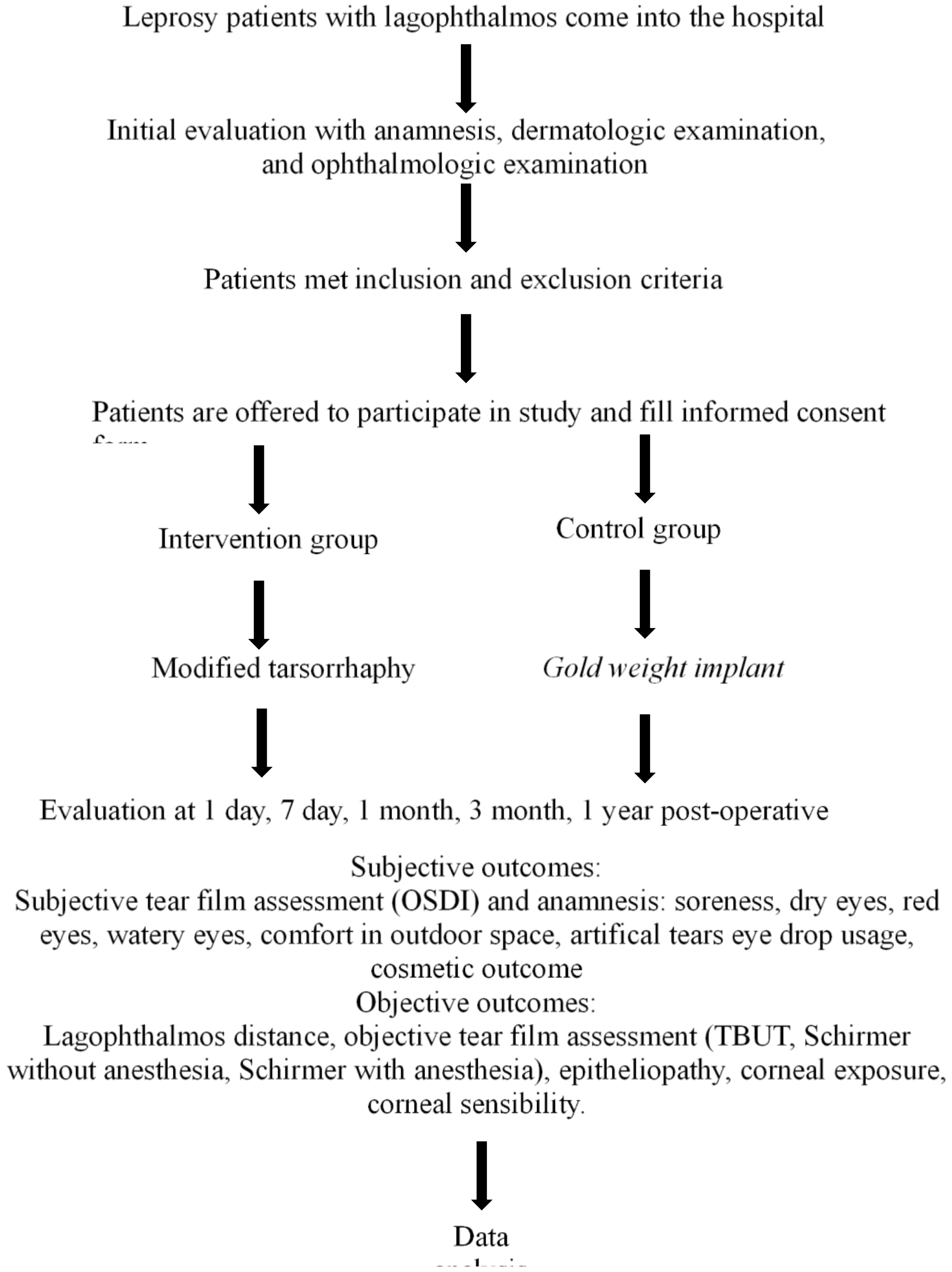

f. Sample Size

This study uses sample size calculation formula for two-tailed hypothesis test:

$$n = \frac{Sd^2 (Z_{1-\alpha/2} + Z_{1-\beta})^2}{(\mu_0 - \mu_a)^2}$$

$$n = \frac{1{,}19^2 (1{,}96 + 0{,}842)^2}{1^2}$$

$$n = 12$$

n = total sample

Sd = standard deviation of mean differennce (1.19)

$Z_{1-\alpha/2}$ = type 1 error (1.96)

$Z_{1-\beta}$ = type 2 error (0.842)

$\mu_0 - \mu_a$ = significant mean difference of both groups (1 mm represents significant)

Researcher determines sample size of minimum 12 eyes in each group.

g. Recruitment

Subjects will be recruited by oculoplastic surgeons as the principal investigator (YI) and research team members (YI, AP, HD) at each hospital centers. To anticipate drop-out, final recruited sample size is added by 20%.

h. Assignment of Interventions

Allocation Sequence Generation

This is a multicenter PROBE (Prospective Randomized Open-label, Blinded-Endpoint) clinical trial study. Research team members will assign treatment received on all sample with randomization using blocking restriction size of 2.

Allocation Concealment Mechanism

Each sample will be assigned a number to conceal allocated intervention.

Implementation

Research assistants (DR, CP) will generate the allocation sequence and assign participants to interventions.

Blinding

Principal investigator (YI) as the oculoplastic surgeon will be informed the intervention assigned to each subject, but the outcomes will be blinded from the principal investigator until the data gathering is completed. Outcomes will be measured by three oculoplastic surgeons with the same qualification as research team members (HD, TR, AP). Unblinding is permissible to the principal investigator if there is any complication occur and subject needs to be treated by the oculoplastic surgeon (as the principal investigator).

3. DATA COLLECTION, MANAGEMENT, ANALYSIS

Data Collection Methods

Data will be collected by research team members (three oculoplastic surgeons with equal qualifications) from the first subject enrolment until last follow up. Data collection instruments include questionnaire, data log, and laboratory tests. Questionnaire and data log are attached in Appendix 2-6.

Data Management

All data will be stored by research assistants (DR, CP) and can only be accessed by them. Principal investigator could access the data after the data collection is completed.

Statistical Methods

Data will be analyzed using univariate, bivariate, and multivariate analysis. Univariate test is used to analyze frequency distribution of study variables. Bivariate test is used to analyze relation between two variables, using Independent T-Test, Wilcoxon, homogenity test, and Mann-Whitney test. Multivariate test uses General Linear Model Repeated Measure with ANOVA. All tests using $\alpha = 0.05$.

4. MONITORING

Any complaints from subjects or adverse events will be informed to the principal investigator or research assistants (contact information is included in the informed consent form). Adverse events will be monitored during study period. Surgical complications will be treated by oculoplastic surgeon. Other complications will be referred to related specialists. Serious adverse events will be judged by research ethics committee.

5. ETHICS AND DISSEMINATION

Research Ethics Approval

This study will be submitted to Medical and Health Research Ethics Committee (MHREC) Faculty of Medicine, Public Health and Nursing Universitas Gadjah Mada - dr. Sardjito General Hospital, Yogyakarta, Indonesia for ethical approval.

Protocol Amendments

Any changes of the protocol will be communicated to relevant parties (research assistants, research team members, investigators, regulators).

Consent

Subjects must sign informed consent form as agreement for participating in this study.

Confidentiality

Subject personal information and study outcomes will be collected and can only be accessed by the research assistants and principal investigator to maintain confidentiality. Data can only be accessed by permission from the principal investigator with prior agreements and not for commercial purposes.

Declaration of Interests

This study is independent from sponsor. All research team members and investigators have no conflict of interest.

Ancillary and Post-Trial Care

Any complaints from subject post-trial can be communicated to the principal investigator or research assistants (contact information is included in the informed consent form).

Dissemination Policy

Investigator plans to submit the result of this study to relevant publication. Full protocol, participant-level dataset, and statistical code can only be accessed with permission and agreement with principal investigator.

APPENDIX 1

INFORMED CONSENT SFOR STUDY CANDIDATES

I, Yunia Irawati, led by myself Yunia Irawati from Faculty of Medicine, Public Health, and Nursing UGM will be doing a study titled " Effectivity and Efficiency Comparison between Modified Tarsorrhaphy and Gold Weight Implant Technique as Operative Management of Paralytic Lagophthalmos in Leprosy Patients".

This study aims to compare effectivity and efficiency between modified tarsorrhaphy technique and gold weight implant technique as operative management of paralytic lagophthalmos in leprosy patients.

Research team offers you to participate in this study. This study needs 26 subjects with more or less of 3 months duration of study.

A. Volunteering to participate in research

You are free to choose to participate in this research without any enforcement. If you have decided to participate, you still have the option to resign or change mind anytime during the study without being subject to any fines or sanctions.

If you are not willing to participate, you are still able to consult the doctor with related specialization.

B. Study Procedure

If you are willing to participate in this study, you are asked to sign this informed consent form in two copies, one for you to save and one for the research team. The next procedure will be:

1. Interview will be conducted by doctor to ask: identity, disease history, mainly about leprosy, history of leprosy medication, and other medications.
2. Filling out questionnaire related to eye subjective symptoms, physical examination, skin examination, simple eye examination with visual acuity, flashlight, and slit lamp.
3. Retinal nerve examination (funduscopy), tear duct/irrigation examination and test using filter paper, examination for sensitivity of the black part of the eyeball (cornea), and examination using dye drop to assess corneal damage. All of them will be examined by research team (ophthalmologist).
4. Patient will be accompanied to Jakarta Eye Center Hospital (Jakarta), Dr. Tadjudidin Chalid Hospital (Makassar), or dr. Cipto Mangunkusumo Kirana Eye Hospital (Jakarta) by research team or research assistant to carry out laboratory tests (Hb, leukocyte, thrombocyte, differential count, blood sugar, bleeding time, clotting time,

HbSAg, albumin protein) as pre-operative preparation, skin scrape test/AFB test with bacterial dye (Ziehl Neelsen).

5. Surgery will be performed by lead researcher, me, as oculoplastic surgeon to reconstruct eyelid with 2 surgery techniques that will be assigned by randomization for each eye.
6. After surgical procedure, medications will be given, such as oral antibiotics, painkiller, eye ointment, and eye drop.

C. Study subject obligation

As study subject, you are obliged to follow the rules or study instructions as written above. If something is not clear, you can ask further more to the researcher. During this study, you have to come to Jakarta Eye Center Hospital (Jakarta), Dr. Tadjudidin Chalid Hospital (Makassar), or dr. Cipto Mangunkusumo Kirana Eye Hospital (Jakarta) at first day, seventh day, first month, third month, and one year after surgery and transportation will be provided by research team.

D. Risk, side effects, and the treatment

Possible post-operative complications are bleeding, infection, granulation, or implant extrusion. However, surgeon will do the best effort with doing surgery in sterile operating room, giving informative education and medication after surgery to minimize post-operative complication. During study period, researcher prepares the necessary protection needed if the unwanted happens. Protection provided by the researcher is subject will be treated according to guidelines at Jakarta Eye Center Hospital (Jakarta), Dr. Tadjudidin Chalid Hospital (Makassar), or dr. Cipto Mangunkusumo Kirana Eye Hospital (Jakarta) by related specialist doctor.

E. Advantages

Direct advantages for the subject are get a laboratory test to check the blood, to know acid-fast bacilli infection in body, and get eyelid reconstruction surgery to repair function and eye appearance with free of charge.

F. Privacy

All informations related to subject identity will be concealed and will only be known by the researcher and assistant researchers. Study result will only be published without subject identities.

G. Compensation

Subject will get eye medications after surgery and direct consultation with ophthalmologist (eye specialist doctor) with free of charge.

H. Financing

All expenses related to the study will be covered by researcher, such as laboratory tests, AFB test, eye examination, gold implant material, surgery cost, and medications after surgery.

I. Additional information

You will be given the opportunity to ask questions related to this study. If at any time side effects occur or subjects need further information, you can contact me at Yunia Irawati, MD contact number +62816789595.

You can also ask about this study to Medical and Health Research Ethics Committee (MHREC) Faculty of Medicine, Public Health and Nursing Universitas Gadjah Mada - Dr. Sardjito General Hospital (phone: 0274-588688 ext 17225 or +62811-2666-869, or email: mhrec_fmugm@ugm.ac.id).

CONSENT TO PARTICIPATE IN RESEARCH STUDY

All explanations have been conveyed to me and all questions have been answered by researcher/doctor. I understand that if I need further information, I can ask to the researcher/doctor.

With signing this form, I agree to participate in this research study

Patient/subject sign: Date:

(Full name: ....)

Witness sign:

(Full name: ....)

APPENDIX 2

RESEARCH STATUS

Date of examination	:		
Research serial number	:		
Medical record number	:		
Name	:		
Date of birth	:	Age	:
Address	:		
	:		
Phone number	:		
Occupation	:	Marital status	:
Gender	: M / F		
Leprosy type	: PB / MB		
Duration of leprosy	:		
Leprosy reaction	: No reaction / Type I Reaction / Type II Reaction		
MDT treatment	: not received yet / in therapy / release from treatment		
Duration of MDT	:		
Corticosteroid treatment	: yes / no		
Duration of corticosteroid	:		
Disability	: none / grade 1 / grade 2		

APPENDIX 4

POST-OPERATIVE EVALUATION

GOLD WEIGHT IMPLANT

Objective	Pre-op		Day - 1		Day - 7		Month - 1		Month - 3		Year - 1	
	OD	OS	OD	OS	OD	OS	OD	OS	OD	OS	OD	OS
Lagophthalmos distance												
Epitheliopathy												
Corneal exposure												
Corneal sensibility												
Schirmer without anesthesia test												
Schirmer with anesthesia test												
Tear Break Up Time test												
Bleeding												
Infection												
Granulation												

APPENDIX 5

POST-OPERATIVE EVALUATION

MODIFIED TARSORRHAPHY

Objective	Pre-op		Day - 1		Day - 7		Month - 1		Month - 3		Year - 1	
	OD	OS	OD	OS	OD	OS	OD	OS	OD	OS	OD	OS
Lagophthalmos distance												
Epitheliopathy												
Corneal exposure												
Corneal sensibility												
Schirmer without anesthesia test												
Schirmer with anesthesia test												
Tear Break Up Time test												
Bleeding												
Infection												
Granulation												

APPENDIX 6

OCULAR SURFACE DISEASE INDEX

**Have you experienced any of the following during the last week?**	**All of the time**	**Most of the time**	**Half of the time**	**Some of the time**	**None of the time**
A. Eyes that are sensitive to light?	4	3	2	1	0
B. Eyes that feel gritty?	4	3	2	1	0
C. Painful or sore eyes?	4	3	2	1	0
D. Blurred vision?	4	3	2	1	0
E. Poor vision?	4	3	2	1	0
**Have problems with your eyes limited you in performing any of the following during the last week?**					
F. Reading?	4	3	2	1	0
G. Driving at night?	4	3	2	1	0
H. Working with a computer or bank machine (ATM)?	4	3	2	1	0
I. Watching TV?	4	3	2	1	0
**Have your eyes felt uncomfortable in any of the following situations during the last week?**					
J. Windy conditions?	4	3	2	1	0
K. Places or areas with low humidity (very dry)?	4	3	2	1	0
L. Areas that are air conditioned?	4	3	2	1	0

OSDI score assessment : Normal: 0 – 12; Mild dry eye: 13 – 22; *Moderate dry eye*: 23 – 32; *Severe dry eye*: 33 – 100
