# Supplementary material for: Modified tarsorrhaphy versus gold weight implant technique for paralytic lagophthalmos treatment in patients with leprosy: One-year observation of a randomized controlled trial study
Source: Front Med (Lausanne). 2023 Jan 4;9:941082. doi: 10.3389/fmed.2022.941082 (PMC9845573; doi:10.3389/fmed.2022.941082)
Supplement: Supplementary file 2 [file Data_Sheet_2.pdf]

Pre-operative picture

Three months post-operative

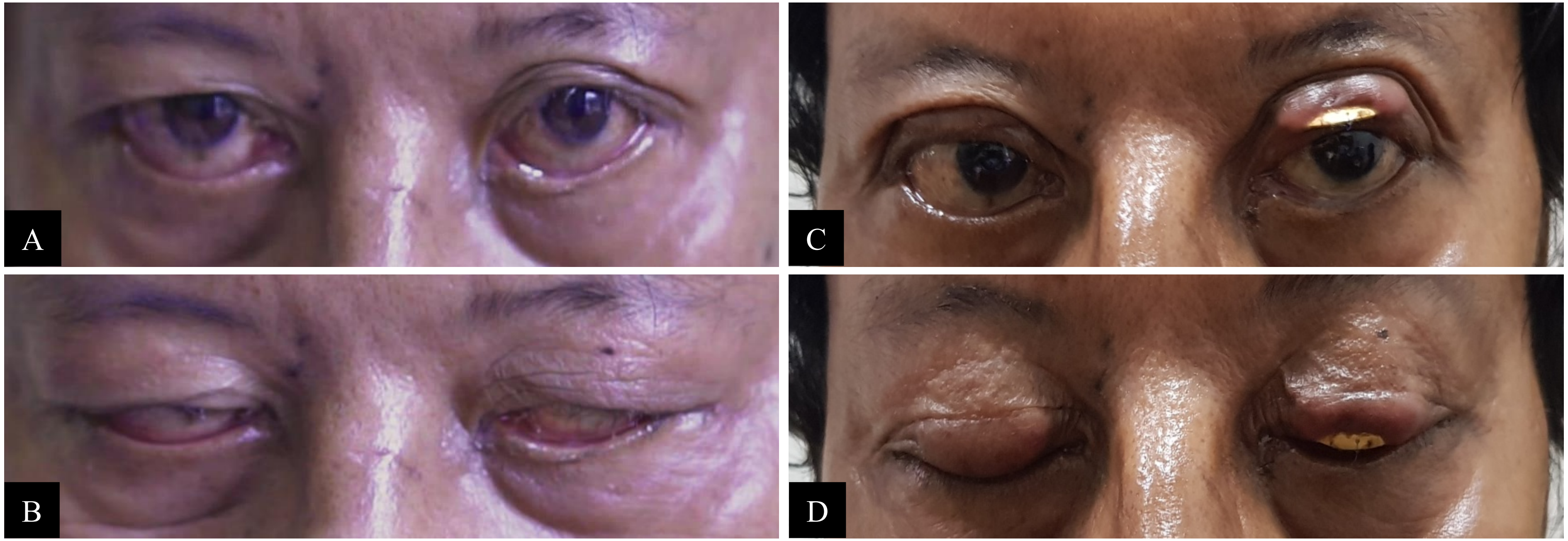

(A) Pre-operative picture of paralytic lagophthalmos in leprosy patient on both eyes opened, and (B) closed showing lagophthalmos distance. (C-D) Three months after gold weight implant procedure on both eyes, the left eye showed implant extrusion.

Pre-operative picture

Three months post-operative

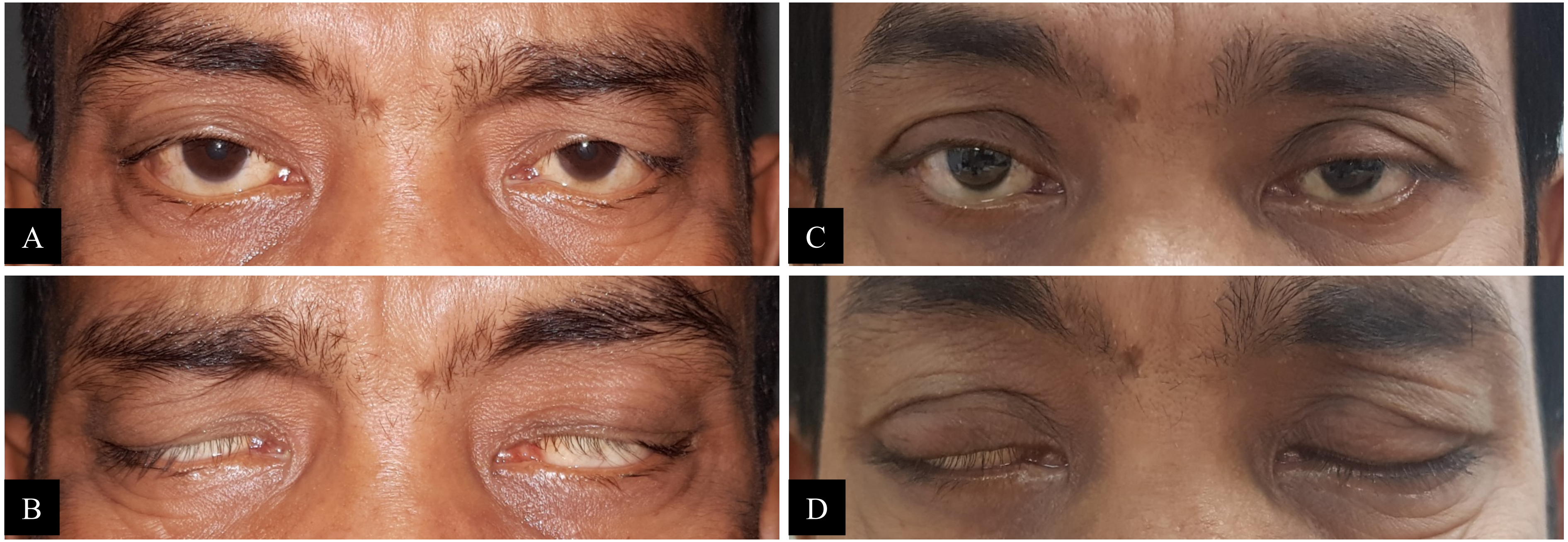

(A) Pre-operative picture of paralytic lagophthalmos in leprosy patient on both eyes opened, and (B) closed showing lagophthalmos distance. (C-D) Three months after modified tarsorrhaphy procedure on both eyes, lagophthalmos distance decreased.

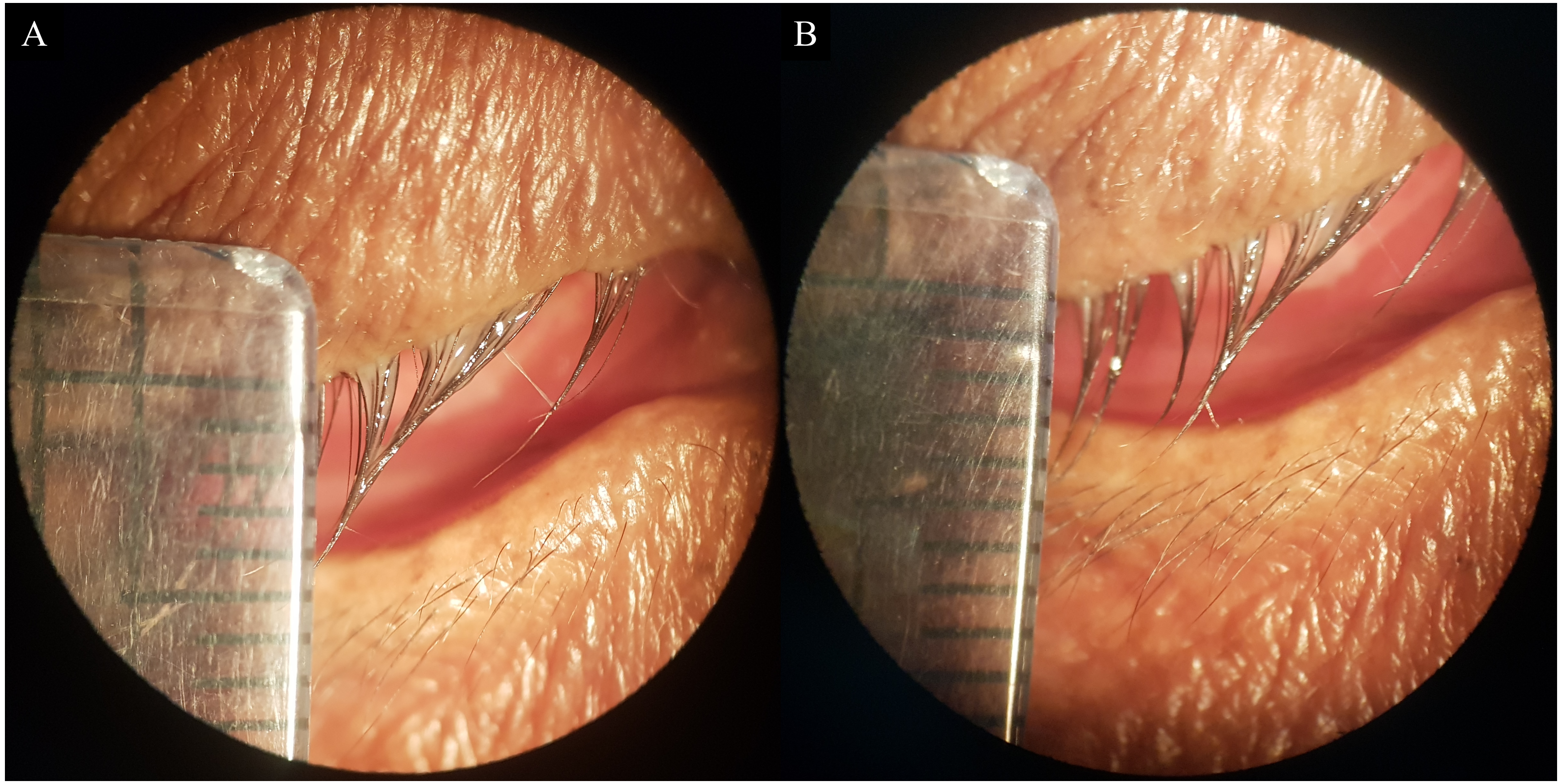

Measuring lagophthalmos distance (A) Central area (B) Temporal area
